# Polycaprolactone Nanoparticles as Promising Candidates for Nanocarriers in Novel Nanomedicines

**DOI:** 10.3390/pharmaceutics13020191

**Published:** 2021-02-01

**Authors:** Sylwia Łukasiewicz, Antoni Mikołajczyk, Ewa Błasiak, Ewelina Fic, Marta Dziedzicka-Wasylewska

**Affiliations:** Department of Physical Biochemistry, Faculty of Biochemistry, Biophysics and Biotechnology, Jagiellonian University, 30-387 Krakow, Poland; antoni.mikolajczyk92@gmail.com (A.M.); ewa.blasiak@uj.edu.pl (E.B.); ewelina.fic@uj.edu.pl (E.F.); marta.dziedzicka-wasylewska@uj.edu.pl (M.D.-W.)

**Keywords:** nanomaterials, polymeric nanoparticles, polycaprolactone, pegylation, macrophage

## Abstract

An investigation of the interactions between bio-polymeric nanoparticles (NPs) and the RAW 264.7 mouse murine macrophage cell line has been presented. The cell viability, immunological response, and endocytosis efficiency of NPs were studied. Biopolymeric NPs were synthesized from a nanoemulsion using the phase inversion composition (PIC) technique. The two types of biopolymeric NPs that were obtained consisted of a biocompatible polymer, polycaprolactone (PCL), either with or without its copolymer with poly(ethylene glycol) (PCL-b-PEG). Both types of synthesized PCL NPs passed the first in vitro quality assessments as potential drug nanocarriers. Non-pegylated PCL NPs were internalized more effectively and the clathrin-mediated pathway was involved in that process. The investigated NPs did not affect the viability of the cells and did not elicit an immune response in the RAW 264.7 cells (neither a significant increase in the expression of genes encoding pro-inflammatory cytokines nor NO (nitric oxide) production were observed). It may be concluded that the synthesized NPs are promising candidates as nanocarriers of therapeutic compounds.

## 1. Introduction

Nanoparticles (NPs) are frequently defined as solid colloidal particles in the range of 10–1000 nm. Polymer nanoparticles (PNPs) are nanospheres and nanocapsules made of polymeric materials [1]. Nanospheres are matrix particles, i.e., particles whose entire mass is solid. Molecules may be adsorbed on the sphere’s surface or encapsulated within the particle matrix. Polymer nanoparticles have recently been growing in importance and they play crucial roles in a wide range of fields, including in electronics, photonics, conducting materials, sensors, medicine, biotechnology, pollution control, and environmental technology [2,3]. Biodegradable nanoparticles are frequently used in medicine and biotechnology to improve the therapeutic value of various drugs. The nanoencapsulation of drugs in PNPs increases their bioavailability, solubility, retention time, efficacy, specificity, tolerability, and drug therapeutic index values [1,3,4,5]. PNPs may be functionalized to achieve so-called “intelligent targeting”, i.e., targeted delivery to specific cells, tissues, or organs [6,7,8]. Numerous biodegradable polymers such as polycaprolactone (PCL), polylactic acid (PLA), polyglycolic acid (PGA), and polylactide-co-glycolide (PLGA) are being tested for possible use in drug delivery systems [9,10]. Several drugs have been successfully encapsulated in PNPs to their improve bioavailability, bioactivity, and control delivery [4,11]. The main applications focus on diseases such as cancer, AIDS, diabetes, malaria, prion disease, and tuberculosis [12,13,14,15,16,17].

The features of PNPs, such as their toxicity, biocompatibility, biodistribution, and immunogenicity, play crucial roles in designing new drug delivery systems [18,19]. It is well known that the dimensions, particle charges, and surface modifications are the parameters that affect these features [20,21]. It is especially important to investigate the impacts of potential nanocarriers on immune system cells, which form the first line of defense against external risks. In particular, it is crucial to create carriers that will be invisible (“the stealth property”) to phagocytic cells, such as the macrophages. Macrophages are cells of the immune system involved in the inflammatory process. Activated macrophages act like scavengers that phagocyte pathogenic molecules. Macrophage activation occurs during their exposure to pathogenic particles, which may also be nanoparticles [22]. This is why the macrophages are the first barrier in the way of drug nanocarriers to their targets, since nanoparticles are seen as alien and are subsequently absorbed and degraded by the phagocytic cells [23]. Therefore, decreasing the uptake of PNPs by the macrophages is one of the main goals in designing new drug delivery systems [24].

One of the most popular and important methods of surface modification that allows the “stealth” properties of PNPs to be increased is the immobilization of a polyethylene glycol (PEG) corona on a particle’s surface. The physicochemical properties of uncharged, hydrophilic polymers, such as their water solubility, extensive hydration, good conformational flexibility, and high chain mobility, cause a steric exclusion effect that provide protein resistance of PEG-based coatings comprising NPs [25,26]. Additionally, PEG side chains enable further functionalization of NPs using specific, well-designed bio-ligands. This strategy leads to greater targeting of the action of the encapsulated drug by transporting it to the site of intended release. For example, folate-decorated NPs carrying a chemotherapeutic agent have been shown to be internalized in cancer cells through FRα-mediated endocytosis [27]. In conclusion, modification of the outer surfaces of NPs by PEG coverings is a key point in controlling the biological fate of pegylated NPs. The process influences the biodistribution, immune system recognition, transport through biological matrices (tumor extracellular matrix, mucus, bacteria biofilm), cellular uptake, and recognition of the destination [27,28].

Generally, PNPs made of polycaprolactone (PCL) attract more attention and are widely studied. To obtained the best nanocarrier designed for targeted drug delivery, it is very important to evaluate its action on many levels. This involves detailed descriptions of the interactions of the carrier with immune cells. Therefore, the present study focuses on investigating PCL NPs and their interactions with phagocytic cells. Copolymers of hydrophilic PEG and hydrophobic PCL typically display high biocompatibility and biodegradability [29]. Grossen at al. [29] reviewed the synthesis, production, description, and application of PEG-PCL-based nanomedicines [29]. In this study, we assess the usefulness of PCL NPs as a potential drug delivery system by describing their interactions with cells of the immune system, namely the RAW 264.7 mouse murine macrophage cell line. Two types of PCL nanoparticles (non-pegylated (PCL) and pegylated (PCL-PEG)) are tested. We investigate the toxicity, immunological influence, and efficiency of macrophage endocytosis. Although the surface PEG length and PEG density are difficult to control, they influence the “stealth” properties of pegylated nanoparticles, as well as their biological behaviors [13,16,17,18,19]. It is well known that NPs pegylated using PEG 5000 demonstrate higher circulation times in the plasma and better distribution in tumor tissue. Moreover, enhanced accumulation in tumor cells has been shown. Qhattal et al. described a nanocarrier that was able to severely inhibit the MDA-MB-231 tumor growth and prolong the survival time of mice harboring B16 tumors [30]

In our study, we use unmodified NPs as well as NPs grafted with a PEG layer. The covalent bonding of polyethylene glycol (PEG) is intended to prevent opsonization by antigens and serum proteins on NP surfaces, thereby increasing the half-life of NPs in the bloodstream by reducing the immune response [6,11]. PEG chains are also more hydrophilic and form a hydration layer around pegylated NPs, making them less efficiently phagocytized by the macrophages. Reducing the NP uptake by the macrophages is one of the main goals in designing new drug delivery systems [8].

## 2. Experimental Section

### 2.1. Chemicals Used for Nanoparticle Preparation

Polymers: Polycaprolactone (PCL) (average Mw ~10 kDa) and poly(ethylene glycol)-block-poly(ε−caprolactone) methyl ether (PCL-b-PEG) (PCL average Mw ~13 kDa, PEG average Mw ~5 kDa), as well as the surfactants TWEEN^®^20 and Span^®^20, were received from Sigma-Aldrich Poland (Poznan, Poland). Solvents: Toluene was purchased from Avantor Performance Materials (Gliwice, Poland), while distilled water was obtained with the Direct-Q 5UV purification system from Millipore (Prospecta Sp. z o.o., Warsaw, Poland). All chemicals were used without further purification.

### 2.2. Materials Used for Cell Culture and Cytotoxicity Tests

The RAW 264.7 mouse murine macrophage cell line was obtained from Sigma-Aldrich Poland (Poznan, Poland). All cell culture materials, including heat-inactivated fetal bovine serum (FBS), DMEM (Dulbecco’s Modiffied Eagle’s Medium, F12 mediums, MTT (3-(4,5-dimethylthiazol-2-yl)-2,5 diphenyl tetrazolium bromide), Triton X-100, and TRI-Reagent, were obtained from Sigma-Aldrich Poland (Poznan, Poland). The lactate dehydrogenase (LDH) cytotoxicity detection kit was purchased from Clontech Laboratories (Thermo Fisher Scientific, Warsaw, Poland. Luminaris HiGreen qPCR (real time PCR) Master Mix and all other molecular reagents were purchased from Thermo Fisher Scientific, Warsaw, Poland. Molecular probe (Thermo Fisher Scientific, Warsaw, Poland)-delivered reagents used for nitrite determination (Griess Reagent Kit) and phagocytosis assay (Vybrant Phagocytosis Kit). The synthesis of oligonucleotides was commissioned at Genomed S.A (Warsaw, Poland).

### 2.3. PCL and PCL-PEG Nanoparticle Preparation

Polycaprolactone (PCL) and pegylated-polycaprolactone (PCL-PEG) nanoparticles were prepared by the nanoemulsion templating method [31]. The nanoemulsion was made using the phase inversion composition (PIC) technique. The phase inversion was achieved by stepwise addition of water to toluene with dissolved polymers (PCL with or without the addition of PCL-b-PEG and a mixture of surfactants (TWEEN^®^20/Span^®^20) at constant room temperature). Following the preparation of the stable nanoemulsion, toluene (a toxic organic solvent) was evaporated and the surfactants were removed by dialysis. To prepare fluorescently labeled nanoparticles, a fluorescent dye (cumarine-6) was encapsulated in the formed nanoparticles. Coumarin-6 was dissolved in toluene (0.5 mg/mL) before the emulsification process. Drug-loaded nanoparticles can also be synthesized, as described previously [31].

### 2.4. PCL and PCL-PEG Nanoparticle Description

The sizes (the hydrodynamic diameters) of the formed nanoemulsion and synthesized nanoparticles were determined using the dynamic light scattering (DLS) technique using a Zetasizer Nano ZS from Malvern Panalytical Instruments (Warsaw, Poland). The values were estimated as averages of at least three subsequent measurements with 20 runs. Additionally, the sizes and concentrations of the synthesized nanoparticles were measured via nanoparticle tracking analysis (NTA) using an NS500 NanoSight instrument from Malvern Panalytical Instruments (Warsaw, Poland). UV-Vis absorption spectra of the loaded nanoparticles, as well as empty ones, were acquired to confirm coumarin-6 encapsulation using the UV-1800 spectrophotometer (Shimadzu) Thermo Fisher Scientific, Warsaw, Poland. To evaluate the stability of the obtained nanoparticles, their sizes were detected over time during storage. The experiments were conducted at 25 °C in the preparation buffer (water), as well as in cell culture media (DMEM containing 10% FBS).

### 2.5. Cell Culture

The mouse murine macrophage cells (RAW 264.7) were cultured in a DMEM medium supplemented with 1% l-glutamine, high glucose, and 10% FBS at 37 °C in a humidified incubator (BINDER (VWR, Radnor, PA, USA) with 5% CO_2_ atmosphere. Two days before the MTT, LDH, and NO experiments, cells at a density of 3 × 10^4^ cells/well were seeded in appropriate 96-well plates. Similarly, two days before flow cytometry and qPCR experiments, the cells were seeded at a density of 3 × 10^5^ cells /well in 6-well plates.

### 2.6. Cell Viability and Cytotoxicity Assays

#### 2.6.1. MTT Reduction Test

To evaluate the RAW 264.7 cell viability, an MTT reduction assay was performed as described previously [32]. For the assay, different types of PCL NPs (pegylated and non-pegylated; doses of ca. (circa) 16,500, 7000, and 3500 NPs per cell) were used. NPs resuspended in a complete fresh medium were added to the appropriate wells and incubated with the RAW 264.7 cells for 24 h. Then, after medium aspiration the cells were treated under standard culture conditions with 50 µL of 0.5 mg/mL MTT (3-(4,5-dimethylthiazol-2-yl)-2,5-diphenyltetrazolium bromide) resuspended in serum-free medium. After 4 h the MTT reagent was eliminated and the cells were incubated and shaken for 10 min with 100 µL of DMSO (dimethyl sulfoxide). The metabolically active cells converted the yellow tetrazolium salt into purple formazan. Data were obtained from absorbance measurements at the wavelength of 570 nm (TECAN Infinitive M200 Pro, TECAN, Männedorf, Switzerland). The RAW 264.7 cells in the control were incubated only with fresh medium containing 0.015 M NaCl. Six replicates were performed for each experiment condition. The final results represent the average cell viability from five independent experiments.

#### 2.6.2. LDH Cytotoxicity Detection Kit

The cytotoxicity detection kit (LDH) was adopted to measure the toxicity of the used nanomaterials as described previously [32]. LDH was detected in the culture medium when the cell membrane was destabilized or destroyed. The assay was run according to the manufacturer’s instructions. Briefly, the RAW 264.7 cells were treated for 4 h with different types of PCL NPs (doses of ca. (circa) 16,500, 7000, and 3500 NPs per cell). Then, after centrifugation (250× *g*, 7 min), 50 µL of the supernatant was incubated for 30 min in the dark at room temperature with 50 µL of the reaction mixture. Finally, the absorbance was recorded at 490 nm (with the reference wavelength at 610 nm) (TECAN Infinitive200, TECAN Männedorf, Switzerland). Spontaneous LDH release was detected from untreated cells (negative control). The maximum LDH level, which corresponds to cell death, was determined after Triton X-100 cell treatment. Six replicates were performed for each experimental condition. The final data exhibiting the cytotoxic potential of the synthesized nanomaterials come from five independent experiments.

### 2.7. Flow Cytometry

The flow cytometry technique was adopted to determine the quantitative cellular uptake as described previously [32]. PCL NPs fluorescently labeled with coumarin-6 were resuspended in fresh full culture medium and added to the cells (doses of ca. (circa) 100, 500, 1000, 2000, 3500, and 5000 NPs per cell). After a 2 h incubation period (37 °C in 5% CO_2_ atmosphere), the medium with PCL NPs was removed and the cells were washed four times with cold phosphate-buffered saline (PBS), pH 7.4). Finally, the cells were suspended in 300 µL of cold PBS and kept on ice until the measurements. The following inhibitors were used to determine the NP endocytosis pathway: chlorpromazine (CPZ 8 µg/mL), filipin III (1 µg/mL), and amiloride (50 µM). Before the experiments, the cells were pre-incubated for 1 h under standard culture conditions with the above-mentioned inhibitors. The PCL NP uptake was determined using a BD FACSCalibur (Becton, Dickinson and Company, San Jose, CA, USA) flow cytometer and CellQuestPro software Becton, Dickinson and Company, San Jose, CA, USA). In total, 10,000 events per sample were acquired. The background fluorescence corresponding to cell autofluorescence was evaluated for the RAW 264.7 cells treated with 0.015 M NaCl or non-fluorescent PCL NPs. Two replicates for each type and dose of the obtained PCL NPs dose were performed. The final data reflect the average of four independent experiments.

### 2.8. NO Determination Test

The Griess Reagent Kit for Nitrite Determination was used to measure the NO release as a result of a 4 h RAW 264.7 treatment with PCL NPs (doses of ca. (circa) 16,500, 7000, and 3500 NPs per cell). The assay was performed according to the manufacturer’s instructions. After 4 h, 75 µL of the medium was transferred to a fresh 96-well plate containing 10 µL of Griess reagent per well. The mixture was incubated for 30 min in the dark at room temperature. The photometric reference sample constituted 10 µL of the Griess reagent and 140 µL of deionized water. The amount of nitrite was evaluated using a spectrophotometric measurement of absorbance at the 548 nm wavelength (TECAN Infinitive200TECAN Männedorf, Switzerland). Untreated cells served as the control. Six replicates for each PCL NP dose were performed. The obtained results represent the average of three independent experiments.

### 2.9. Visualization Studies

Fluorescence microscopy was used for the visualization of the RAW 264.7 cells after 2 h PCL NP (fluorescently labeled with coumarin-6) treatment (ca. (circa) 2500 NPs per cell). Two days before the experiment, the RAW 264.7 cells were seeded on 10 mm plates at a density of 1 × 10^5^ cells per well. Images were acquired using an EVOS fluorescence microscope (Life Technologies, Carlsbad, CA, USA) (Thermo Fisher Scientific, Warsaw, Poland) with 480 nm excitation and 520 nm emission.

### 2.10. Quantitative Real-Time PCR Experiments

Two days before the experiment, the RAW 264.7 cells were seeded on 6-well plates at a density of 3 × 10^5^ cells per well. The PCL NPs were added in various doses (ca. (circa) 2000, 3500, AND 5000 NPs per cell) to the complete fresh medium in each well and incubated at 37 °C in a 5% CO_2_ incubator for 4 h. TRI Reagent and phenol–chloroform extraction were used for the total RNA isolation. After reverse transcription of 1 µg of total RNA, the obtained cDNA was used in the qPCR reaction. For this kind of experiment, the specific primers and Luminaris HiGreen qPCR Master Mix (Eco™ Real-Time PCR System, Illumina, Cambridge, UK) were used. Untreated cells were used as the reference samples, whilst GAPDH (D-Glyceraldehyde 3-phosphate:NAD) served as the reference gene. Below is the list of specific primers used for qPCR reactions: GAPDH-for (TCAACGGCACAGTCAAGG), GAPDH-rev (ACTCCACGACATACTCAGC), IĸBαFOR (CTTGGTGACTTTGGGTGCTGAT), IĸBαREV (GCGAAACCAGGTCAGGATTC), iNOFOR (TCCTACACCACACCAAAC), iNOREV (CTCCAATCTCTGCCTATCC), IL-6FOR (TTCTCTGGGAAATCGTGGAAA), TNF-αFOR (CCCTCACACTCAGATCATCTTCT), TNF-αREV (GCTACGACGTGGGCTACAG).

## 3. Results and Discussion

**Figure 1:**
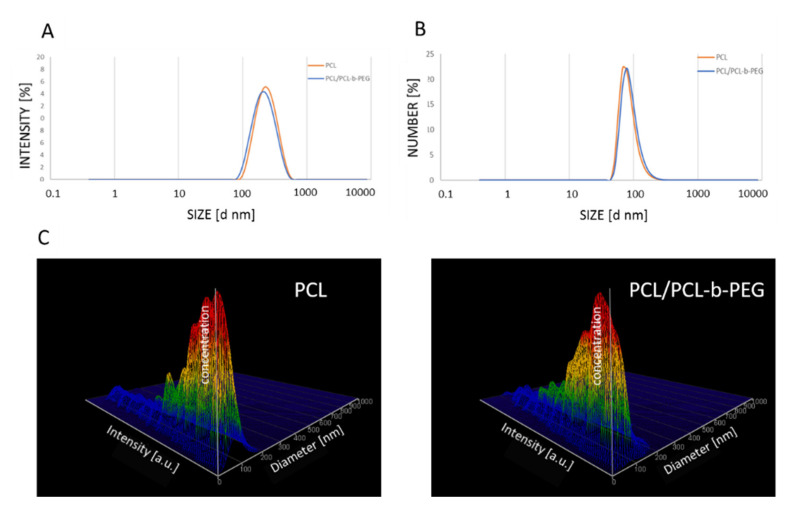
(**A**) Size distribution of nanoemulsion droplets containing PCL (orange) and PCL/PCL-b-PEG (blue), with an average size of ~250 nm. Size distribution of the synthesized PCL and PCL/PCL-b-PEG nanoparticles measured using the DLS (**B**) and NTA (**C**) techniques. Abbreviations: PCL (polycaprolactone), PEG (poly(ethylene glycol), DLS (dynamic light scattering), NTA (nanoparticle tracking analysis).

PCL nanoparticles (both non-pegylated (PCL) and pegylated (PCL-PEG)) were prepared using the nanoemulsion templating method as described previously [31]. At the beginning, a stable nanoemulsion containing selected polymers and actives was prepared using the PIC technique. The nanoemulsion was formed via drop-by-drop addition of water to the polymer solution, containing PCL (3 mg mL^−1^) or PCL with PCL-b-PEG (2.88 mg mL^−1^ and 0.13 mg mL^−1^, respectively) and a mixture of non-ionic surfactants (TWEEN^®^20/Span^®^20) in toluene. The formed nanoemulsion contained: 20% (*v*/*v*) oil phase, 5% (*v*/*v*) TWEEN^®^20/Span^®^20 (HLB = 13.5), and 75% (*v*/*v*) water. The mean size of the nanoemulsion droplets containing PCL and PCL/PCL-b-PEG (PCL-PEG) measured as by the dynamic light scattering (DLS) technique was ~250 nm with a polydispersity index (PdI) value < 0.2, as shown in Figure 1A.

In the end toluene, was evaporated with a rotary evaporator, which led to the formation of polymeric nanoparticles. The mixture of surfactants (TWEEN^®^20/Span^®^20) was removed using dialysis. The average size of the prepared PCL and PCL-PEG nanoparticles measured by DLS or NTA was ~90 nm, with a Pdi value of below 0.2 (Figure 1B,C).

**Figure 2:**
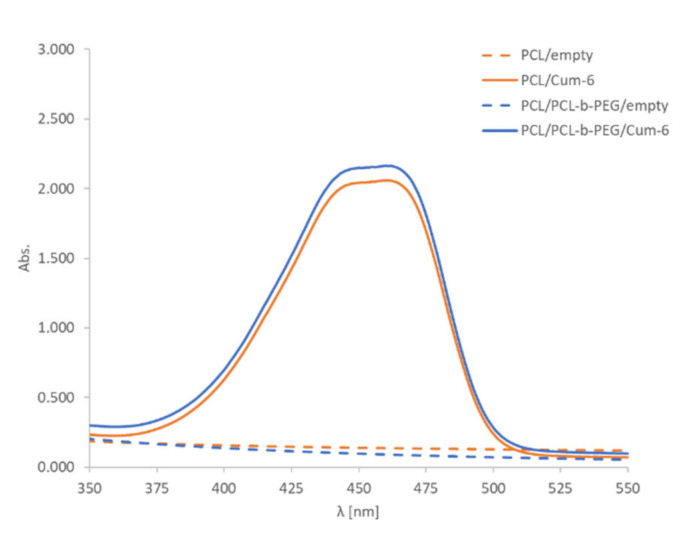
The UV-Vis spectra of PCL and PCL/PCL-b-PEG nanoparticle (~2 × 10^11^ nanoparticles/mL) water suspensions containing coumarin-6 (Cum-6). Water was the solvent used for UV-vis spectra.

The encapsulation of the model active substance, i.e., the fluorescent dye coumarin-6, was confirmed by UV-Vis spectrophotometry analysis. The comparison of the spectra for empty polymeric and coumarin-6-loaded nanoparticles provided evidence of successful encapsulation of the drug. A characteristic peak at 460 nm in the UV-Vis spectra of the nanoparticle suspension containing coumarin-6 was observed (Figure 2). The final concentration of PCL and PCL-PEG nanoparticles as measured using the NTA technique was ~2 × 10^11^ nanoparticles/mL. A nanosystem’s biocompatibility and long-term stability are important parameters for its potential biomedical application. The stability of nanoparticles in DMEM containing 10% fetal bovine serum (FBS) was evaluated, and we found that they retained their size without showing any significant changes for at least 48 h. Synthesized nanoparticles (PCL and PCL-PEG) were formed with bio-acceptable components, except for toluene, which was evaporated after preparation.

**Figure 3:**
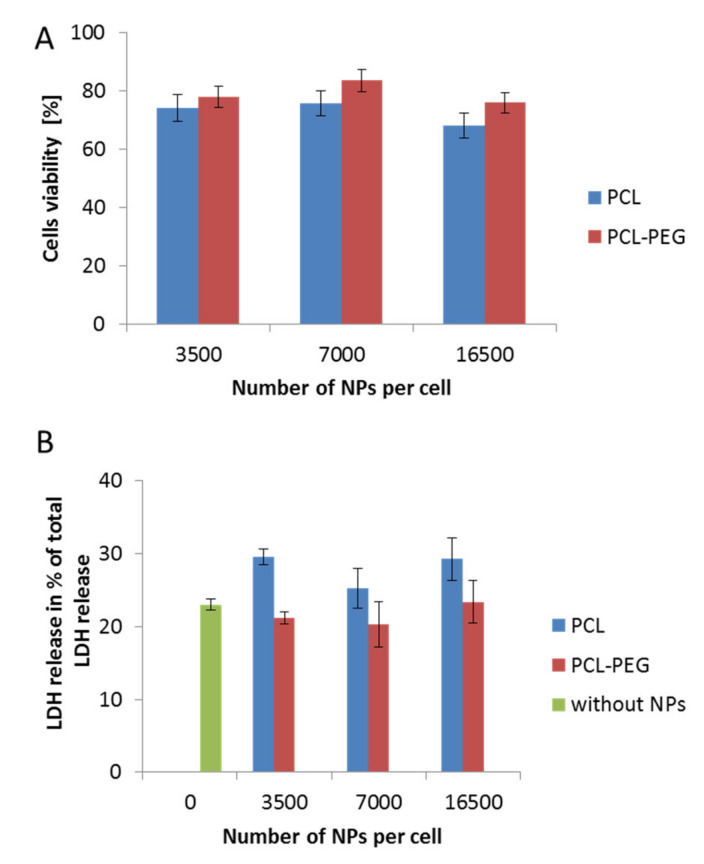
Biocompatibility studies performed in RAW 264.7 cells, showing the influence on cell stability of various types of synthesized PCL NPs used in different doses. (**A**) MTT cell viability assay after a 24 h incubation period with NPs. (**B**) Cytotoxicity assay showing LDH (Lactic Dehydrogenase) release (over a 4 h incubation period with NPs). Data represent the ± standard error of the mean (SEM).

The determination of the interactions between nanocarriers and certain model cell lines is a crucial step in designing a nanoparticulate system for controlled drug delivery. Estimating the possible toxicity of nanomaterials is very important and must be taken into consideration in the first step of the investigation. Therefore, to evaluate changes in cell viability following incubation with the obtained PCL NPs, various assays were conducted. The results obtained using the MTT and LDH tests were consistent (Figure 3A,B). They indicated a safer action profile of pegylated PCL NPs. In both cases (PCL with or without the PEG layer), we observed no changes related to cell membrane disruption, which probably resulted from the negative surface charge of the used PCL NPs. A previous study indicated the contribution of positively charged molecules to the toxicity of nanomaterials due to membrane disruption [33].

As indicated by the previous studies, NPs are quickly eliminated from the bloodstream after injection [23,34]; therefore, we investigated the interactions of different types of synthesized PCL nanoparticles with phagocytic cells (the RAW 264.7 cell line). It is well known that cells of the mononuclear phagocytic system (MPS) such as macrophages have the potential to recognize and remove NPs before they reach their destination. Plasma protein adsorption on the surfaces of NPs plays a key role in this process [19].

This phenomenon leads to a reduction of the circulating half-life, and hence affects the capacity of nanomaterials to be efficient nanovehicles in a controlled drug transportation system. Moreover, it has been shown that the macrophage response depends on the particle size and surface charge [19,35]. It appears that the NP size affects the cellular uptake rate and the internalization mechanism [36]. Modification of nanocarrier surface properties by “stealth” polymers, e.g., by PEG, leads to a deceleration of the opsonization process, which consequently increases the half-life of the NPs in the bloodstream. Moreover, “stealth” polymers also protect NPs by suppressing their uptake by the macrophages [37]. It has been shown that the higher protein adsorbability of hydrophobic compared to hydrophilic surfaces enables the uptake of more hydrophobic particles by the phagocytes in vitro, as well as the quick clearance of hydrophobic particles in vivo [38]. A significant role in prevention of protein adsorption and cell membrane disruption can be achieved through modification of the surfaces of NPs by covering well-hydrated PEG chains. The process masks the original surfaces of NPs and provides steric hindrance. This effect is correlated with the PEG properties. Proper pegylation of the particle surfaces is a crucial step, as the PEG quality, chain size, number of chains, density, and the way they are arranged have huge impacts on the interactions with the target cells and biodistribution of the nanocarrier in the body [39]. Wang et al. studied the effects of surface PEG length on in vivo delivery of PCL NPs, finding that NPs with a PEG surface length of 13.8 nm (MW = 5000 Da) significantly decreased the absorption of serum protein and interactions with macrophages, which finally translated into increased blood circulation time, enhanced tumor accumulation, and improved antitumor efficacy [26].

**Figure 4:**
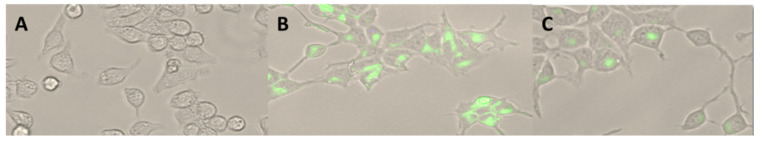
RAW 264.7 cells (**A**) after a 2 h incubation period with PCL NPs (**B**) and PCL-PEG NPs (**C**) (green), which were assessed using fluorescence microscopy. 30× magnification.

Visualization using fluorescent microscopy allowed observation of the presence of PCL NPs in the cytoplasm of the RAW 264.7 cells, showing that internalization of PEG-modified NPs differed remarkably in comparison to unmodified NPs (Figure 4).

**Figure 5:**
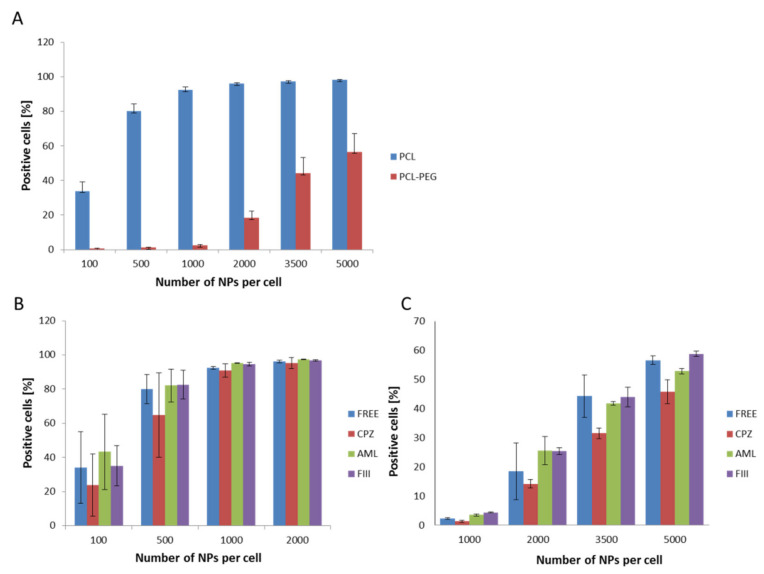
Internalization assessment of various types of PCL NPs by the RAW 254.7 cells (flow cytometry measurements). (**A**) Measurements for different NP doses (**B**,**C**), showing the influence of specific endocytosis inhibitors (CPZ-chlorpromazine, AML-amiloride, FIII-filipino III) on NPs internalization. The results obtained for CPZ were significantly different (Student’s *t* test) from the control (non-treated cells). (**B**) Non-pegylated PCL NPs. (**C**) Pegylated PCL NPs. Data represent the ± standard error of the mean (SEM).

The first set of uptake experiments sing flow cytometry conducted at 4 °C confirmed that the internalization process was active, as significant inhibition of the endocytosis process was recorded. The following experiments show the efficiency of PCL NP internalization by the RAW 264.7 cells. For both types of PCL NPs, a positive correlation between the endocytosis level and the administered PCL NP dose was determined (Figure 5A). Non-pegylated PCL NPs were internalized more effectively and we observed the saturation of cells from a dose of 1000 NPs per cell. In the case of PCL-PEG NPs, we observed a significant reduction of endocytosis (Figure 5A). As mentioned above, prevention of opsonization is crucial in order to reduce the efficiency of the recognition and capture of nanocarriers by the cells of the immune system. The obtained results indicate that PCL NP coating with a layer of highly hydrated PEG chains significantly slows down the absorption of PCL NPs by the macrophage cells. This is definitely a desired effect, especially considering the fact that the potential carrier should be less visible to the cells of the immune system. The presented results are consistent with our previous studies carried out using polymeric nanocapsules of another type [32,39,40,41,42]. Recent studies [43] showed that doxorubicin (DOX)-loaded PCL-PEG NPs modified by collagenase IV (ColIV) and clusterin (CLU) were effectively accumulated in MCF-7 tumor cells and at the same time they overcame the phagocytosis by RAW264.7. Moreover, the interaction of DOX-loaded mPEG-PCL NPs grafted by 2-hydroxyethyl cellulose (HEC) with macrophages cells indicates that such particles are not recognized as foreign bodies [44].

The available literature data point to the engagement of various endocytosis pathways in the NPs internalization process [45]. Therefore, in the present study the internalization mechanism of the synthesized PCL NPs was investigated. Before the experiment, the RAW 264.7 cells were pre-incubated with specific agents that abolish the defined endocytosis pathway. Chlorpromazine (CPZ) causes the formation of clathrin-coated pits [46] and was used to inhibit clathrin dependent endocytosis. Amiloride activity is connected with blocking of the Na^+^/H^+^ exchanger and the prevention of membrane ruffling, and thus it inhibits micropinocytosis [47,48]. Caveolae-dependent internalization is diminished by filipin III, an inhibitor that binds to cholesterol and distorts the structure and functions of cholesterol-rich membrane domains [47]. Data obtained in the study indicate that the clathrin-mediated endocytosis pathway (Figure 5B,C) is engaged in the internalization of PCL NPs.

**Figure 6:**
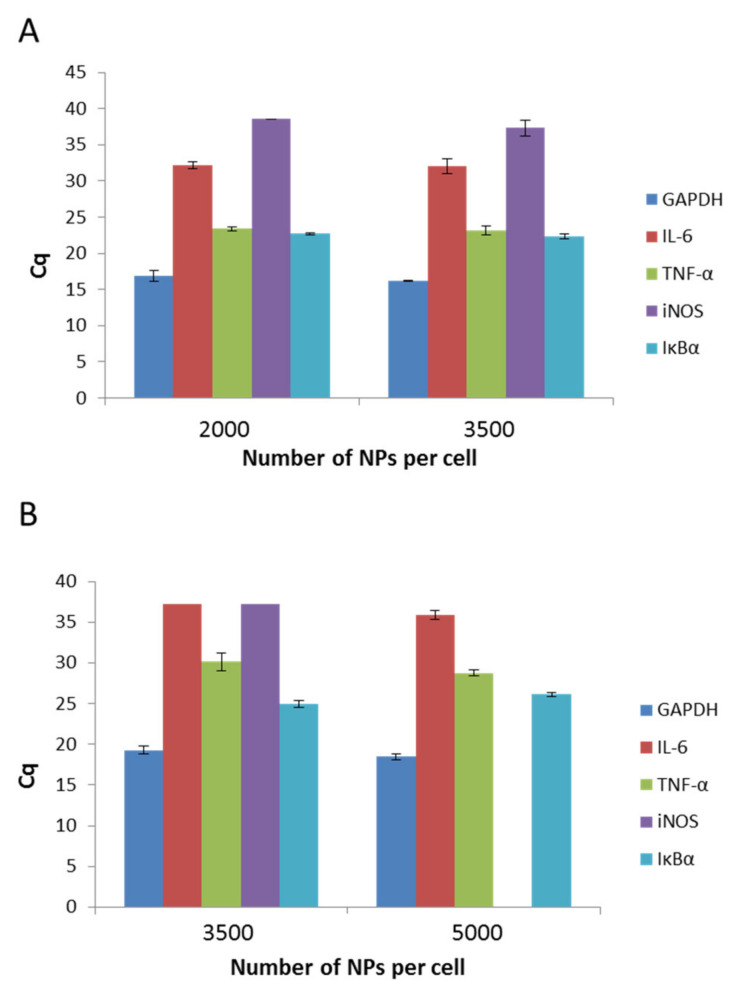
Expression levels of IL-6 (interleukin 6), TNF-a (tumor necrosis factor), iNOS (induced nitric oxide synthase), and IkBa (nhibitory protein for the factor NF-kB), as determined by Cq value. (GAPDH 3-phosphoglycerate dehydrogenase) serves as a control): (**A**) after a 4 h incubation period with non-pegylated NPs; (**B**) after a 4 h incubation period with pegylated NPs. Data represents the ± standard error of the mean (SEM).

The physicochemical parameters of nanomaterials affect their behavior in organism systems. Therefore, in the present study, we aimed to determine if the presence of both types of PCL NPs induces inflammation in the RAW 264.7 cell line. To exclude the pro-inflammatory activity of PCL NPs, we performed qPCR experiments, which detect the levels of interleukin 6 (IL-6), tumor necrosis factor (TNF-α), induced nitric oxide synthase (iNOS), and inhibitory protein for the factor NF-kB (IĸBα). GAPDH (3-phosphoglycerate dehydrogenase) was the reference gene. The outcomes were analyzed against a reference sample, which constituted non-NP treated cells. As we observed no expression (except TNF-α) of the investigated genes in the reference sample, data estimation based on the Rq method was impossible and the results of the experiments are shown using the Cq value, which reflects the fluorescence signal originating from particular genes exceeding the threshold value. A recap of the experiment is presented in Figure 6A,B. It is evident that for both kinds of PCL NPs, regardless of the used doses, the levels of two genes (TNF-α and IĸBα) increased. TNF-α is a key factor that is indispensable in the proper functioning of macrophages and other immune cells. The cytokine plays an important role in proliferation, maintenance of cellular homeostasis, and tumor progression. Its importance in modulating immunological processes is also well known [49]. Therefore, the similar levels of detection for both NP treated and untreated cells were not surprising. The second gene that was expressed in the cells was the gene for IĸBα. A major function of IĸBα is to bind with the NF-kB, and thus to suppress its pro-inflammatory activity. The result obtained for IL-6 is similarly significant. This well-known major pro-inflammatory cytokine was not detectable in our experiments. To conclude, bearing in mind the influence of PCL NPs on the expression of immune mediators in the RAW 264.7 cells, its use as a nanovehicle for active compounds may be considered.

**Figure 7:**
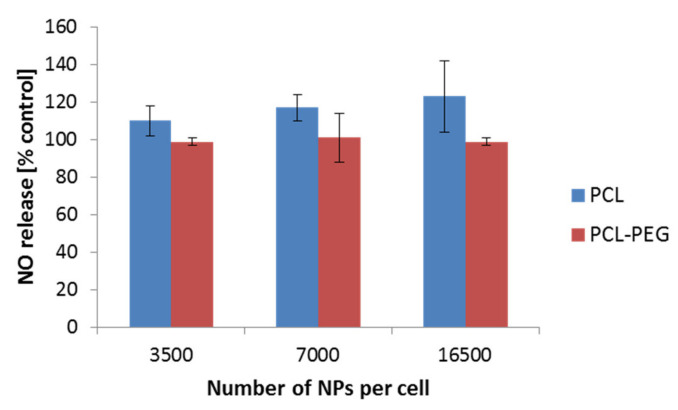
NO detection after a 4 h incubation period of the RAW 264.7 cells with PCL NPs. Data represent the ± standard error of the mean (SEM).

Nitric oxide (NO) is well known for its various functions. Its engagement in the immunological response has been widely described. Therefore, we additionally focused on the determination of the NO level as a result of the interaction of PCL NPs with the RAW 264.7 cell line. We did not observe a significant increase of the NO level in the performed experiments (Figure 7). Similar results were observed by Abamor et al. for J774 macrophages cells [50]. Taken together with the qPCR experiments, the obtained results suggest low ability of the tested PCL NPs to induce an immune response.

## 4. Conclusions

PCL NPs have been used in a few studies, however they must be thoroughly characterized before they can be used in vivo. Therefore, any new experimental design that allows better insight into the interactions of NPs with cells could be considered novel. There are many studies on novel nanomedicine applications, especially in the field of nanocarriers, however very often the studies are focused on investigating the activities of drugs released in target destinations. For example, in some tumor tissues there have been very good descriptions of how these drugs act, their interactions with tumor cells, and how efficient the delivery process is, however unfortunately these studies lack insight into how the loaded nanocarriers behave in the whole organism, which in our opinion is very important. One has to consider the following issue—what will we achieve if we destroy cancer cells and cause an excessive immune response that threatens a patient’s life?

The determination of the interactions between nanocarriers and living model cell lines is a prerequisite to designing a nanoparticulate system for controlled drug delivery. Our studies showed that both of the synthesized PCL nanoparticles (non-pegylated (PCL) and pegylated (PCL-PEG)) passed the first in vitro quality assessments as potential drug nanocarriers. The tested nanoparticles did not affect the viability of the tested cells and did not elicit a response from the immune system (in experiments with the RAW 264.7 cells). Non-pegylated nanoparticles were preferentially uptaken by the tested cell line rather than the pegylated ones. Based on our results, it may be concluded that the synthesized PCL and PCL-PEG nanoparticles are promising candidates for nanocarriers of therapeutic compounds. Their potential clinical use should be the subject of future studies.

## Data Availability

The data presented in this study are available on request from the corresponding author. The data are not publicly available due to no requirements in this respect on the part of the research financing project.
